# Multimodality quantitative ultrasound envelope statistics imaging based support vector machines for characterizing tissue scatterer distribution patterns: Methods and application in detecting microwave-induced thermal lesions

**DOI:** 10.1016/j.ultsonch.2024.106910

**Published:** 2024-05-17

**Authors:** Sinan Li, Po-Hsiang Tsui, Weiwei Wu, Zhuhuang Zhou, Shuicai Wu

**Affiliations:** a Department of Biomedical Engineering, College of Chemistry and Life Sciences, Beijing University of Technology, Beijing, China; b Department of Medical Imaging and Radiological Sciences, College of Medicine, Chang Gung University, Taoyuan, Taiwan; c Division of Pediatric Gastroenterology, Department of Pediatrics, Chang Gung Memorial Hospital at Linkou, Taoyuan, Taiwan; d Liver Research Center, Chang Gung Memorial Hospital, Linkou, Taoyuan, Taiwan; e Research Center for Radiation Medicine, Chang Gung University, Taoyuan, Taiwan; f College of Biomedical Engineering, Capital Medical University, Beijing, China

**Keywords:** Quantitative ultrasound, Machine learning, Multimodality ultrasound envelope statistics imaging, Tissue scatterer distribution, Ultrasound tissue characterization

## Abstract

Ultrasound envelope statistics imaging, including ultrasound Nakagami imaging, homodyned-K imaging, and information entropy imaging, is an important group of quantitative ultrasound techniques for characterizing tissue scatterer distribution patterns, such as scatterer concentrations and arrangements. In this study, we proposed a machine learning approach to integrate the strength of multimodality quantitative ultrasound envelope statistics imaging techniques and applied it to detecting microwave ablation induced thermal lesions in porcine liver *ex vivo*. The quantitative ultrasound parameters included were homodyned-K α which is a scatterer clustering parameter related to the effective scatterer number per resolution cell, Nakagami *m* which is a shape parameter of the envelope probability density function, and Shannon entropy which is a measure of signal uncertainty or complexity. Specifically, the homodyned-K $\log_{10}(\alpha )$, Nakagami-*m*, and horizontally normalized Shannon entropy parameters were combined as input features to train a support vector machine (SVM) model to classify thermal lesions with higher scatterer concentrations from normal tissues with lower scatterer concentrations. Through heterogeneous phantom simulations based on Field II, the proposed SVM model showed a classification accuracy above 0.90; the area accuracy and Dice score of higher-scatterer-concentration zone identification exceeded 83% and 0.86, respectively, with the Hausdorff distance <26. Microwave ablation experiments of porcine liver *ex vivo* at 60–80 W, 1–3 min showed that the SVM model achieved a classification accuracy of 0.85; compared with single $\log_{10}(\alpha ),m$, or hNSE parametric imaging, the SVM model achieved the highest area accuracy (89.1%) and Dice score (0.77) as well as the smallest Hausdorff distance (46.38) of coagulation zone identification. We concluded that the proposed multimodality quantitative ultrasound envelope statistics imaging based SVM approach can enhance the capability to characterize tissue scatterer distribution patterns and has the potential to detect the thermal lesions induced by microwave ablation.

## Highlights

• A multi-QUS envelope statistics imaging SVM method for scatterer distribution.
• Ultrasound log_10_(*α*), *m*, and hNSE as the input features of the SVM classifier.
• Phantom simulations and MWA experiments of porcine liver *ex vivo* were conducted.
• The proposed method outperformed single QUS envelope statistics imaging method.

## 1 Introduction

Quantitative ultrasound (QUS) is a frontier ultrasound technology that can measure quantified information related to tissue microstructures [1], [2], [3], [4], [5]. Characterization of tissue microstructures is important because when a tissue undergoes a pathological change or therapy, a microstructural alteration may occur. In the context of QUS, tissue microstructures can be characterized by extracting quantitative parameters connected with acoustic scatterers from ultrasound backscattered signals, as opposed to the commonly used B-mode ultrasound which is qualitative. The QUS parameters frequently used in ultrasound tissue characterization include backscatter coefficient [1], [2], [4], [6], acoustic attenuation [1], [2], [4], speed of sound [4], [5], envelope statistics [1], [2], [3], [4], [6], [7], [8], [9], [10], [11], Lizzi–Feleppa spectral parameters [1], [2], [12], [13], [14], mean scatterer spacing [15], [16], [17], scatterer number densities [18], [19], and scatterer sizes [7], [20], [21].

Ultrasound envelope statistics imaging is an important group of QUS techniques, including ultrasound Nakagami imaging, homodyned-K (HK) imaging, and information entropy imaging, as they can characterize scatterer distribution patterns [3], [6], [7], [8], [9], [10], [11], [22], [23], [24], [25], [26], such as local scatterer concentrations and arrangements, which are useful in disease diagnosis, therapy evaluation or monitoring, and prognosis prediction. Ultrasound Nakagami and HK imaging methods are model-based envelope statistics imaging techniques, where the Nakagami or HK models are used for describing the probability density functions (PDFs) of the envelopes of ultrasound radiofrequency (RF) signals. The Nakagami [27], [28] and HK [29], [30], [31] distributions are generalized statistical models for ultrasound backscatter envelope statistics [31]. The Nakagami distribution has two parameters, *m* and Ω, which can describe the shape and scale of envelope PDFs, respectively. The shape parameter *m* is connected with scatterer concentrations and arrangements. The HK distribution has three parameters of physical meanings, α,ε, and σ, where α is the scatterer clustering parameter related to the effective number of scatterers per resolution cell, $\varepsilon^{2}$ denotes the coherent signal power, and $2\alpha \sigma^{2}$ represents the diffuse signal power [31]. A derived parameter of the HK distribution, *k*, can describe the ratio of the coherent to diffuse signal, where $k=\varepsilon /(\sigma \sqrt{\alpha })$. It should be noted that the Nakagami distribution can be considered as an approximation of the HK distribution [31]. Ultrasound information entropy imaging methods are non–model-based envelope statistics imaging techniques, where entropy provides an insight into RF signal uncertainty and allows for the analysis of backscattered statistics without considering the distribution of data or the statistical properties of ultrasound signals [1]. A histogram is usually used to estimate the probability value of each histogram bin, and these probability values are used to calculate the Shannon entropy. It has been demonstrated that ultrasound Shannon entropy imaging can reflect the changes of scatterer concentrations [32].

Thermal tumor ablation, such as microwave ablation (MWA) and radiofrequency ablation (RFA), is a minimally invasive therapy technique [33], where an applicator is inserted into a tumor to heat the tumor, causing coagulative necrosis of the tumor cells to kill the tumor *in situ*. High-intensity focused ultrasound (HIFU) does not need an applicator and enables heating of a target tissue volume from sources placed outside the body which produce a focused ultrasound field. The heating induced gas bubbles and tissue necrosis in a coagulation zone or ablation zone causes tissue microstructural changes which can be reflected in scatterer distribution patterns. These bubbles can play the role of “natural” contrast agents, contributing to additional acoustic scatterers in the ablation zone. The interaction of the bubbles with incident ultrasound waves results in additional signal components in the RF signals [34], [35], [36]. Ultrasound envelope statistics imaging techniques have shown potential in detecting or monitoring heating-induced thermal lesions [11], [22], [23], [24], as they are capable to characterize local scatterer distribution alterations in the ablation zone, such as the formation and growth of heating-induced bubbles. Zhang et al. [37], [38] explored the feasibility of using Nakagami imaging to evaluate the ablation zone induced by HIFU exposures in tissue-mimicking phantoms. Huang et al. [39] conducted *ex vivo* porcine liver HIFU experiments to compare Nakagami imaging to conventional B-mode ultrasound imaging in identifying thermal lesions. Wang et al. [40] demonstrated that Nakagami imaging could visualize thermal lesions of porcine liver *ex vivo* induced by RFA. Zhou et al. [41] proposed a real-time Nakagami imaging method based on frequency and temporal compounding for monitoring RFA and measuring the areas of ablation zones in porcine livers and muscle tissues *ex vivo*. Zhang et al. [42], [43] demonstrated that Nakagami imaging had potential to monitor and characterize thermal lesions induced by MWA through *ex vivo* and *in vivo* porcine livers experiments. Our previous study [22] presented an algorithmic scheme of Nakagami imaging based on Gaussian pyramid decomposition, with MWA coagulation zone detection based on polynomial approximation (PAX). Our previous studies demonstrated the feasibility of HK imaging in monitoring thermal lesions in porcine livers *ex vivo* induced by MWA [11], [24]. Monfared et al. [44] demonstrated that Shannon entropy imaging could detect thermal lesions in porcine muscle tissue *ex vivo* induced by HIFU, and exhibited higher lesion-to-normal contrast comparing to the Nakagami imaging. Li et al. [45] proposed a horizontally normalized Shannon entropy (hNSE) imaging method for monitoring MWA-induced thermal lesions of porcine livers *ex vivo* and *in vivo*.

In this study, we focused on QUS envelope statistics imaging as it is one of well-established QUS techniques associated with tissue microstructures. Although ultrasound Nakagami imaging, HK imaging, and information entropy imaging methods have been used for characterizing tissue scatterer distribution patterns, an approach that integrates the strength of multimodality envelope statistics imaging techniques have not been investigated, particularly in terms of MWA. We hypothesized that using a machine learning model to combine the features of ultrasound Nakagami imaging, HK imaging, and information entropy imaging may enhance the capability to characterize tissue scatterer distribution patterns. In this study, we proposed a support vector machine (SVM) approach to combining multimodality QUS envelope statistics imaging techniques for characterizing tissue distribution patterns. We conducted simulation experiments and *ex vivo* porcine liver MWA experiments to evaluate the performance of the proposed method. The major contributions of this work are as follows.

• A multimodality QUS envelope statistics imaging based SVM method for tissue scatterer distribution characterization was proposed.
• Ultrasound HK-$\log_{10}(\alpha )$, Nakagami-*m*, and hNSE parameters were extracted form parametric maps to construct the feature vectors, which were inputs of the SVM classifier.
• Phantom simulations and MWA experiments of porcine liver *ex vivo* were conducted to evaluate the performance of the proposed method.
• The proposed method was compared to single QUS envelope statistics imaging method with adaptive threshold segmentation in identifying the boundary of coagulation zones.

## 2 Materials and methods

### 2.1 Phantom simulations

The heterogeneous phantom simulations were conducted using the ultrasound simulation toolbox Field II [46], [47]. A 3.5-period Gaussian excitation pulse (central frequency = 7.5 MHz) was used as the incident wave and a 192-element linear-array transducer with 32 active elements was used to generate backscattered echo signals. Acoustic attenuation and noise were neglected. The width, elevation height, and kerf of the element was 0.3 mm, 4 mm, and 12.5 μm, respectively. The speed of sound was 1540 m/s and the sampling frequency was set at 30 MHz. The central frequency and sampling frequency were set to conform to the physical transducer in the *ex vivo* experiments. The three-dimensional (3D) resolution cell corresponding to the smallest resolvable detail was considered as the full width at half maximum of the point spread function in the axial, lateral, and elevation directions, which was estimated using the method in [48]. A relatively rough approximation was used in this paper to regard the volume of resolution cell as a cube with the dimensions equal to each resolution [49]. Thus, we obtained a 3D resolution cell of 0.3713 ${\mathrm{mm}}^{3}$ (i.e., 0.7175 mm × 0.7194 mm × 0.7194 mm), and generated various phantoms according to different number densities of scatterers per resolution cell. The volume of the heterogeneous phantoms was x×y×z (lateral × elevational × axial) = 40 mm × 1.5 mm × 40 mm, and there was a cylindrical dense scattering inclusion with a diameter of 10 mm in the center. A total of five types of heterogeneous phantoms were generated. When the scatterer concentration of the background media was 2 scatterers/resolution cell, the scatterer concentrations of the inclusion media were 16, 24, and 32 scatterers/resolution cell, respectively. When the scatterer concentration of the background media was 10 scatterers/resolution cell, the scatterer concentrations of the inclusion media were 32 and 64 scatterers/resolution cell, respectively. Thirty groups of experiments were conducted for each type of heterogeneous phantoms. Each group of experiments yielded a frame of backscattered signals, corresponding to a single phantom. The backscattered signals with a size of 1558 (sampling points) × 256 (scan lines) were obtained from each phantom. The inclusion with higher scatterer concentrations and the background with lower scatterer concentrations were used to simulate different scatterer concentrations. In this study, a SVM classifier was used to classify these two scattering media.

### 2.2 MWA experiments of porcine liver *ex vivo*

**Fig. 1:**
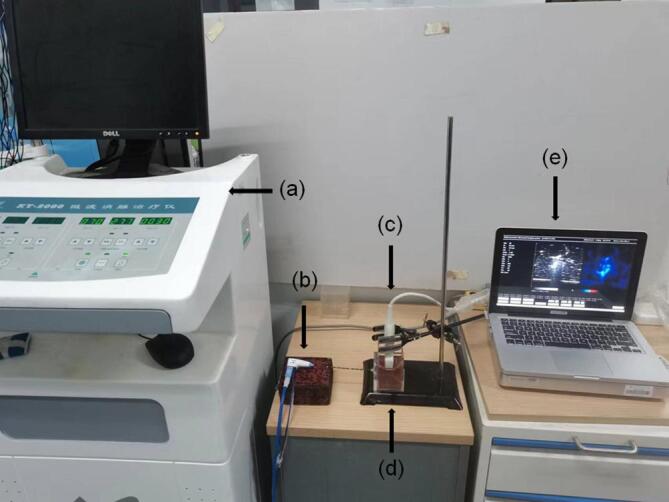
Experimental setup for microwave ablation and ultrasound backscattered signal acquisition. (a) microwave ablation device; (b) microwave ablation needle; (c) linear-array transducer; (d) polycarbonate box; (e) ultrasound scanner.

**Fig. 2:**
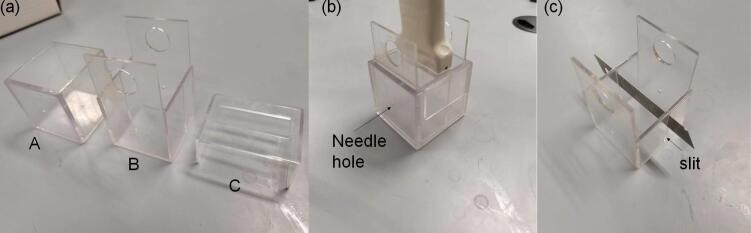
The polycarbonate box for holding porcine liver samples *ex vivo*. (a) It consists of three components: A. Outer box, B. Inner box, and C. Upper cover. (b) The assembled object of three components. The ultrasonic transducer just happened to be stuck by the opening left by the upper cover. (c) There were slits with a width of 0.5 mm left on both sides of the inner box where a blade could be used to slice from top to bottom.

We conducted 60 cases of MWA experiments of porcine liver *ex vivo* (*n* = 60) at 80 W for 1 min (*n* = 20), 70 W for 2 min (*n* = 20), and 60 W for 3 min (*n* = 20). Porcine livers slaughtered and freshly excised within 12 h were purchased from a local market, and they were immersed in 0.9% saline before the experiments. Fig. 1 shows the experimental setup for MWA and ultrasound backscattered signal acquisition. The thermal lesions were induced in porcine livers *ex vivo* using a clinical MWA system (KY-2000, Nanjing Kangyou Co., Ltd., Jiangsu, China). Before cutting the porcine liver sample into an appropriate shape and placing it in the polycarbonate box (5 cm × 5 cm × 5 cm), the B-mode image was used to avoid large vessels or cavities in the area to be ablated [45]. Fig. 2 shows the polycarbonate box for holding porcine liver samples *ex vivo*. Fig. 2(a) shows that the polycarbonate box consists of three components: A. Outer box, B. Inner box (just enough to fit into the outer box without leaving any gaps), and C. Upper cover (with an opening of 18 mm × 50 mm, and just enough to cover the outer box without leaving any gaps). Fig. 2(b) shows the assembled object of three components. A water-cooled MWA needle (KY-2450B) was inserted into the sample horizontally through the circular hole with a diameter of 2 mm left on the side of the outer and inner boxes, and a linear-array transducer (12L5A, Terason) with a central frequency of 7.5 MHz [pulse length (PL) = 0.7 mm] just happened to be stuck by the opening left by the upper cover and caused the transducer surface to sink to a certain distance from the inner box (in order to nearly contact the porcine liver samples). The insertion depth of the ablation needle was measured and set in advance, and the transducer scanned vertically from top to bottom on the plane each time where the central heating point of the needle tip was located. During the ablation, ultrasound backscattered data [1558 (sampling points) × 256 (scan lines)] were acquired by a C++ program [41] developed in-house on the ultrasound scanner (Model 3000, Terason, Burlington, MA, USA) at a sampling frequency of 30 MHz and saved as.bin format files at 2 frames/s for off-line processing. For each case of experiments, only the frame corresponding to the end time of MWA duration was used in this study. After the MWA, the transducer and upper cover were removed, and the ablation needle was pulled out. The inner box was then taken out from the outer box using the handle. There were slits with a width of 0.5 mm left on both sides of the inner box (on the scanning plane of the transducer) [Fig. 2(c)]. The porcine liver samples were sliced by a blade from top to bottom in the inner box (corresponding to the imaging plane through the center of the lesion) in preparation for gross pathology photograph.

### 2.3 Ultrasound envelope statistics parameter estimation and imaging

The two-dimensional envelope data were obtained from backscattered signals using the Hilbert transform. A sliding window with a specific window side length (WSL) was used to traverse the entire envelope data to obtain the parametric maps. For the local envelope data in each window, a specific estimator was used to calculate the parameter value, which was assigned to a new pixel located in the center of the window. A 50% overlap was set for the sliding window and cubic spline interpolation was adopted to make the parametric map have the same size as the envelope. Finally, the parametric images were obtained by using pseudo-color mapping. Specifically, each element in the resized parametric map was assigned a specific color through the MATLAB colormap() function, so that the color of a pixel in the yielded parametric image corresponded to a specific value of the envelope statistics parameter. A colormap was a matrix of values that define the colors for images and consisted of three columns. Each row in the matrix defines one color using an RGB (red, green, blue) triplet. An RGB triplet is a three-element row vector whose elements specify the intensities of the red, green, and blue components of the color. We used the MATLAB colormap(jet) function to gradually change the pixel color from blue to cyan, green, yellow, and finally to red, indicating the variation of parameter values from 0 to 1. In addition, the B-mode image can be yielded by logarithmic-compression and grayscale mapping of the envelope data.

For the selection of the estimators for envelope statistics parameters, we considered fundamental ones that have been frequently used in the field of QUS, because the focus of this study was to evaluate the feasibility of multimodality envelope statistics imaging based SVMs in enhancing scatterer distribution pattern characterization. Specifically, the XU estimator [50] was used for HK parameter estimation because of its low root mean square error, the moment-based estimator [28] was utilized for Nakagami parameter estimation because of its high computational efficiency, and the probability distribution histogram (PDH) based estimator [45] was adopted for hNSE parameter estimation because of its sensitivity to disorder of data.

#### 2.3.1 The HK parameter estimation

Under the HK distribution, the PDF of envelope data is modeled as [31], [50]

(1) $$f_{\mathrm{HK}}(A|\varepsilon ,\sigma^{2},\alpha )=A\int_{0}^{+\infty }{\mathrm{uJ}}_{0}(u\varepsilon )J_{0}(\mathrm{uA}){\left(1+\frac{u^{2}\sigma^{2}}{2}\right)}^{-\alpha }\mathrm{du},$$

where A⩾0 denotes the envelope amplitude; α>0 denotes the scatterer clustering parameter and reflects the effective number of scatterers per resolution cell; $\varepsilon^{2}$ (ε⩾0) and $2\alpha \sigma^{2}$ (σ>0) denote coherent and diffuse signal powers, respectively; $\mu =\varepsilon^{2}+2\alpha \sigma^{2}$ is the mean intensity; and $J_{0}(.)$ denotes the Bessel function of the first kind, zeroth order. The HK-α parameter was demonstrated to be feasible as a parametric imaging and was better than the HK-*k* parameter (i.e., the ratio of the coherent to diffuse signal) for MWA thermal lesion monitoring [24]. From the experiments, we found that the value of the HK-α parameter had a large dynamic range and parametric imaging of HK-$\log_{10}(\alpha )$ had a better visualization capability than that of HK-α, so HK-$\log_{10}(\alpha )$ was used as a QUS feature input to the SVM model in this study. The estimation method of HK distribution parameters based on the mean intensity and *X*- and *U*-statistics, namely the XU estimator, was used in this study [50]:

(2) $$\mu =\overset{¯}{I}\text{;}$$

(3) $$X=\overset{‾}{I\log I}/\overset{‾}{I}-\overset{‾}{\log I}\text{;}$$

(4) $$U=\overset{‾}{\log I}-\log\overset{‾}{I},$$

where $I=A^{2}$ is the intensity of envelope signals. Given a sample set of envelope signals $\left\{A_{1},\ldots ,A_{N}\right\}$, the notation $\overset{‾}{g(A)}$ represents the mean value of the function g(.) over the sample set. For instance, $\overset{¯}{I}$ is equal to $\frac{1}{N}\sum_{i=1}^{N}A_{i}^{2}$. In order to solve the nonlinear system of equations for the variables $\varepsilon^{2},\sigma^{2}$, and α, it would be convenient to adopt an algorithmic parameter $\gamma ,\gamma =\varepsilon^{2}/(2\sigma^{2})$. Consequently, the analytical expressions $X_{\mathrm{HK}}(\alpha ,\gamma )$ and $U_{\mathrm{HK}}(\alpha ,\gamma )$ for the two log-moments as a function of the model parameters were obtained, and the estimation was formulated as a minimization problem [50]:

(5) $$\begin{array}{cc} (\varepsilon^{2},\sigma^{2},\alpha )=\mathrm{argmin}(\left|U_{\mathrm{HK}}-U\right|), & s.t.:\;\;\;\mu =\overset{¯}{I}\text{;}X_{\mathrm{HK}}=X\text{;}\alpha ⩽\alpha_{\max} \end{array}$$

where $\alpha_{\max}$ denotes the upper bound of α ($\alpha_{\max}$ = 40.5 in this work), and the subscript ‘HK’ denotes analytic (i.e., theoretical) values. The parameter α which optimally satisfies the equations were estimated using a binary search algorithm [50]. In this study, four times the transducer PL [11] was adopted as the WSL to obtained the HK-$\log_{10}(\alpha )$ parametric map.

#### 2.3.2 The Nakagami-*m* parameter estimation

The Nakagami distribution has an envelope PDF as [28]:

(6) $$f_{\mathrm{Nak}}(A)=\frac{2m^{m}}{\Gamma (m)\Omega^{m}}A^{2m-1}\exp(-\frac{m}{\Omega }A^{2}),$$

where Γ(.) denotes the gamma function; *m* is the shape parameter, and Ω is the scale parameter. It has been shown that the variation of *m* parameter between 0 and 1 could describe the PDF of envelope amplitude ranging from pre-Rayleigh to Rayleigh distributions [28]. This characteristic allows *m* parametric imaging to detect the variation of local scatterer concentrations in a tissue [51]. It was demonstrated that a sliding window of 3 PLs is sufficient to obtain stable parameter estimation [51]. In this study, the *m* parameter was estimated by the moment-based estimator [28]:

(7) $$m=\frac{{\left[E(A^{2})\right]}^{2}}{E{\left[A^{2}-E(A^{2})\right]}^{2}},$$

where E[.] denotes the expectation operator.

#### 2.3.3 The hNSE parameter estimation

The Shannon entropy of the backscattered envelope signal *r* can be estimated in a discrete form [52]:

(8) $$H_{T}=-\overset{\mathrm{bins}}{\underset{i=1}{\sum }}w(r_{i})[\log_{2}w(r_{i})]=-\overset{r_{\max}}{\underset{r=r_{\min}}{\sum }}w(r)\cdot \log_{2}[w(r)],$$

where w(r) denotes the probability distribution of envelope data, which can be calculated using the PDH. The bin number, bins, was set to 60 in accordance with the phantom simulations and *ex vivo* experiments. The PDH consists of bins that divide the bin limits $\left[r_{\min},r_{\max}\right]$ into equal intervals, so *r* and w(r) are the center and height of bins, respectively. The height of a bin depends on the number of data falling into the bin divided by the total amount of envelope data. For the hNSE parameter estimation, ultrasound backscattered envelope data were normalized into [0, 1] through

(9) $$r\prime =(r-r_{\min})/(r_{\max}-r_{\min})\text{.}$$

The bin limits of the hNSE estimator are always [0, 1], rather than varying with envelope data. Therefore, the entropy value was estimated by [45]

(10) $$H_{N}=-\overset{1}{\underset{r\prime =0}{\sum }}w(r\prime )\cdot \log[w(r\prime )]\text{.}$$

A sliding window with the WSL of 1 PL [32] was adopted to construct the hNSE parametric images.

### 2.4 Feature extraction and classifier training

**Fig. 3:**
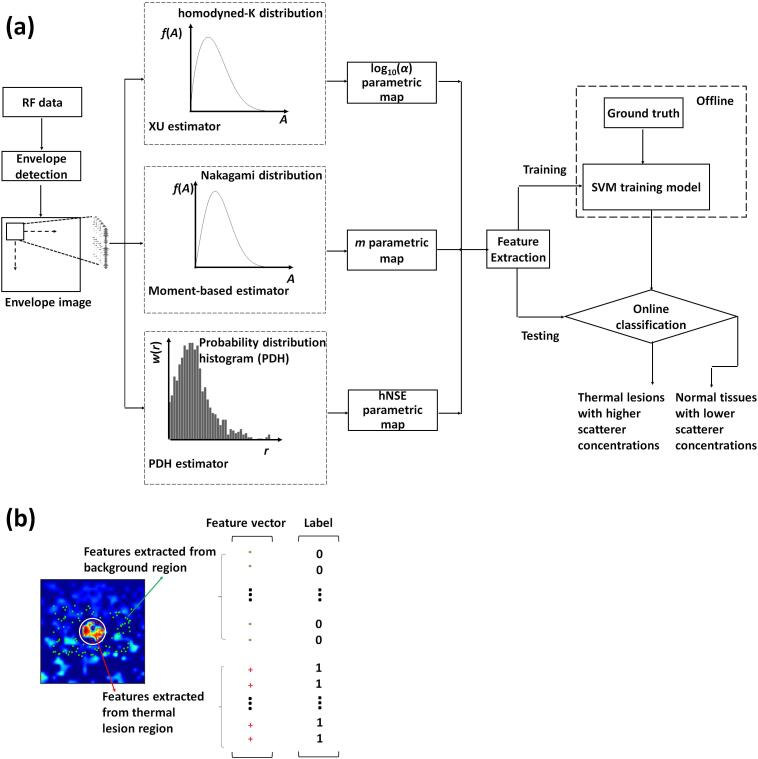
(a) The block diagram for the quantitative ultrasound-based multi-parameter support vector machine (SVM) classifier for training and predicting (testing). Ultrasound backscattered signals are preprocessed to obtain homodyned-K $\log_{10}(\alpha )$, Nakagami-*m*, and horizontally normalized Shannon entropy (hNSE) maps. Thus, each input backscattered signal matrix can provide feature combinations [$\log_{10}(\alpha ),m$, hNSE] $\in R^{3}$ as the inputs of the SVM classifier. With the training sets, SVM constructs the optimal hyperplane to separate two classes: thermal lesions and normal tissues. (b) Illustration of features extracted from each parametric map, that is, randomly selecting 100 pixel points from the target (thermal lesions, indicated by red crosses) and background (normal tissues, indicated by green points) regions, respectively. Each parametric map provides a feature vector containing 200 points, where the points from the background region are labeled as 0, and the points from the target region are labeled as 1.

The QUS-based multi-parameter SVM classifier algorithm scheme is shown in Fig. 3(a). In the preprocessing step, the HK-$\log_{10}(\alpha )$, Nakagami-*m*, and hNSE parametric maps were obtained through the estimation methods described above. Then, each parametric map was normalized to [0, 1] by

(11) $${\mathrm{Para}}_{\mathrm{norm}}=(\mathrm{Para}-{\mathrm{Para}}_{\min})/({\mathrm{Para}}_{\max}-{\mathrm{Para}}_{\min}),$$

where *Para* is the value of a pixel in a parametric image; ${\mathrm{Para}}_{\mathrm{norm}}$ is the normalized value of the pixel; and ${\mathrm{Para}}_{\max}$ and ${\mathrm{Para}}_{\min}$ are the maximum and minimum values of the image, respectively. Instead of using the images directly as the machine learning input, this study used a feature combination of three QUS parameters as the input: [HK-$\log_{10}(\alpha )$, Nakagami-*m*, and hNSE] $\in R^{3}$. This feature vector was used in this study because we experimentally found that it was optimal. (See the Discussion section, i.e., Section 4, for the selection of input features.) The illustration of feature extraction is shown in Fig. 3(b). For each parametric map, one hundred pixel values were randomly selected from the target (thermal lesions) and background (normal tissues) regions, respectively. Thus, each backscattered signal matrix provided 200 feature combinations. In other words, the feature combination extracted from each input backscattered signal matrix is described as ${\left\{\left(x,y\right)\right\}}_{N=1}^{N=200}$, where x = [$\log_{10}(\alpha ),m$, hNSE] $\in R^{3}$, and y is the class corresponding to each set of features, and there are two classes: thermal lesions (y=1), and normal tissues (y=0).

For the simulated phantom data, feature combinations were randomly selected from 20 of the 30 experiment groups for each of the three types of inclusion scatterer concentrations to construct the training set, so the total number of feature combinations was 4000. Note that each group corresponded to one frame of data. For the data collected from MWA experiments of porcine livers *ex vivo*, feature combinations were selected randomly from 45 of 60 cases (i.e., 15 cases were selected for each combination of treatment power and duration) to form the training set, so the total number of feature combinations was 9000. We used the LIBSVM toolbox [53] to train a SVM classifier. The SVM classifier was trained using Gaussian radial basis functions and a sequential minimal optimization (SMO) algorithm [54] to classify the two classes of data. The soft margin parameter *C* and kernel parameter γ should be set for model training, which were optimized using a grid search method [55]. A 10-fold cross validation was performed to find the optimal hyperplane for classification. Briefly, the training set was randomly divided into 10 equally sized subsamples. The classifier was trained on 9 of the sample sets and tested on the remaining sample set. The process was then repeated 10 times. The accuracy of cross validation was computed and the optimal *C* and γ parameters were obtained when the accuracy was the highest. The optimal *C* and γ parameters were used to obtain the final classification model.

### 2.5 Classifier performance evaluation

**Fig. 4:**
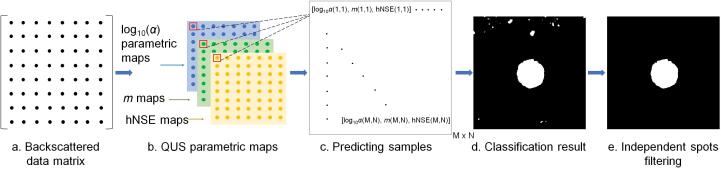
Illustration of the workflow of quantitative ultrasound-based multi-parameter classification and imaging.

The remaining 10 groups of five separate phantoms and 15 cases of MWA data were used to provide testing samples to evaluate the performance of the SVM classification model. Fig. 4 shows the process of testing sample construction and classification. Unlike the training set, the testing samples were constructed pixel by pixel using the estimated three QUS parametric maps, that is, the HK-$\log_{10}(\alpha )$, Nakagami-*m*, and hNSE parameters corresponding to the same pixel point were composed into a feature combination according to the original size of ultrasound backscattered data matrix. Then, the classifier predicted the classes of the feature combinations pixel by pixel into a 0/1 label, and a binarized image was obtained. The performance of the classifier models was evaluated by classification accuracy *ACC*, sensitivity *SEN*, specificity *SPE*, and the area under receiver operating characteristic (ROC) curve (AUC):

(12) $$\mathrm{ACC}=(\mathrm{TP}+\mathrm{TN})/\left(\mathrm{TP}+\mathrm{FN}+\mathrm{TN}+\mathrm{FN}\right)\text{;}$$

(13) $$\mathrm{SEN}=\mathrm{TP}/\left(\mathrm{TP}+\mathrm{FN}\right)\text{;}$$

(14) $$\mathrm{SPE}=\mathrm{TN}/\left(\mathrm{TN}+\mathrm{FP}\right),$$

where *TP* (true positive) and *FP* (false positive) denote prediction classes that were judged as thermal lesions by the classifier whereas in fact they were thermal lesions and normal tissues, respectively. *FN* (false negative) and *TN* (true negative) denote prediction classes that were judged as normal tissues by the classifier whereas in fact they were thermal lesions and normal tissues, respectively. The ROC curve analysis was performed using the MATLAB perfcurve() function, in which the AUC is equal to the probability that a classifier will rank a randomly chosen positive instance higher than a randomly chosen negative one [56].

### 2.6 Thermal lesion detection

After obtaining classification results using the QUS-based SVM classifier in Fig. 4(d) for testing samples, binarized segmentation images were yielded. In general, the thermal lesion region should be an independent connected region, because the temperature field caused by power radiation was a continuous field during MWA [57]. Therefore, independent spots would be filtered out and holes of predicted ablated regions would be filled. To this end, the MATLAB imfill() function was used to fill holes in the classification results [i.e., the binarized image in Fig. 4(d)], and the MATLAB regionprops() function was used to compute the areas of each connected component in the image. Then, the maximum connected region in the final segmentation image was regarded as the thermal lesion region [Fig. 4(e)], and the smaller connected components were all deleted. In addition, binarized segmentation images were also obtained from HK-$\log_{10}(\alpha )$, Nakagami-*m*, and hNSE parametric imaging by the Otsu algorithm [58] to compare with the proposed multi-parameter SVM classifier in terms of the performance of thermal lesion detection. Since Otsu [58] is a non-parametric and unsupervised method of automatic threshold selection for image segmentation, we took advantage of its simple and convenient calculation to achieve thermal lesion detection rapidly. In each binarized image, the target (ablated) region was denoted by 1, and the background (non-ablated) region was denoted by 0.

### 2.7 Thermal lesion detection performance evaluation

We used the ImageJ software (National Institutes of Health, USA) to outline the thermal lesion region from the photo of the tissue section (gross pathology) and binarized it into a mask as the classification ground truth. The images after binarized segmentation, spots filtering, and hole filling were resized to the same pixel size of the mask so that the area detection accuracy of thermal lesions could be calculated as [22]

(15) $${\mathrm{ACC}}_{\mathrm{area}}=\left(1-\frac{\left|{\mathrm{Area}}_{\mathrm{truth}}-{\mathrm{Area}}_{\mathrm{detected}}\right|}{{\mathrm{Area}}_{\mathrm{truth}}}\right)\times 100\%,$$

where ${\mathrm{Area}}_{\mathrm{truth}}$ is the ground truth area of thermal lesions obtained from the mask, and ${\mathrm{Area}}_{\mathrm{detected}}$ is the predicted area calculated from the binarized image, which was obtained from multi-parameter SVM classifier or single parametric imaging. Besides, the Dice score *DS* [59] and Hausdorff distance *HD* [60] were used to evaluate the similarity between the finally obtained binarized images (i.e., the predicted region of thermal lesions) and the ground truth (the actual region of thermal lesions), which were calculated as

(16) $$\mathrm{DS}=\frac{2\mathrm{TP}}{2\mathrm{TP}+\mathrm{FP}+\mathrm{FN}}\text{;}$$

(17) $$\mathrm{HD}(I_{\mathrm{pred}},I_{\mathrm{truth}})=\underset{a\in I_{\mathrm{pred}}}{\max}\{\underset{b\in I_{\mathrm{truth}}}{\max}[\mathrm{dist}(a,b)]\},$$

where *a* and *b* are pixels of the predicted binarized image $I_{\mathrm{pred}}$ and the ground truth image $I_{\mathrm{truth}}$, respectively, and dist(a,b) is the Euclidean distance. The larger the *DS* (with the maximum of 1) and the smaller the *HD* (with the minimum of 0), the higher the similarity between the prediction and the ground truth.

The signal processing, parameter estimation, SVM training and testing, and the calculation of evaluation indicators in the five types of phantom simulations and MWA experiments were all implemented offline using MATLAB R2020b (MathWorks, Natick, MA, USA).

### 2.8 Statistical analysis

The statistical analysis of evaluation indicators was implemented using IBM SPSS Statistics 20 (IBM Corp., NY, USA). A Shapiro–Wilk normality test was performed on area accuracies, Dice scores, and Hausdorff distances to determine whether they followed a normal distribution [61]. One-way analysis of variance (ANOVA) test [62] and Kruskal–Wallis test [63] were used to analyze the difference in means for normally distributed groups and difference in medians for non-normally distributed groups, respectively. Thus, we checked whether there were significant differences between the proposed method and single QUS envelope statistics imaging methods.

## 3 Results

**Fig. 5:**
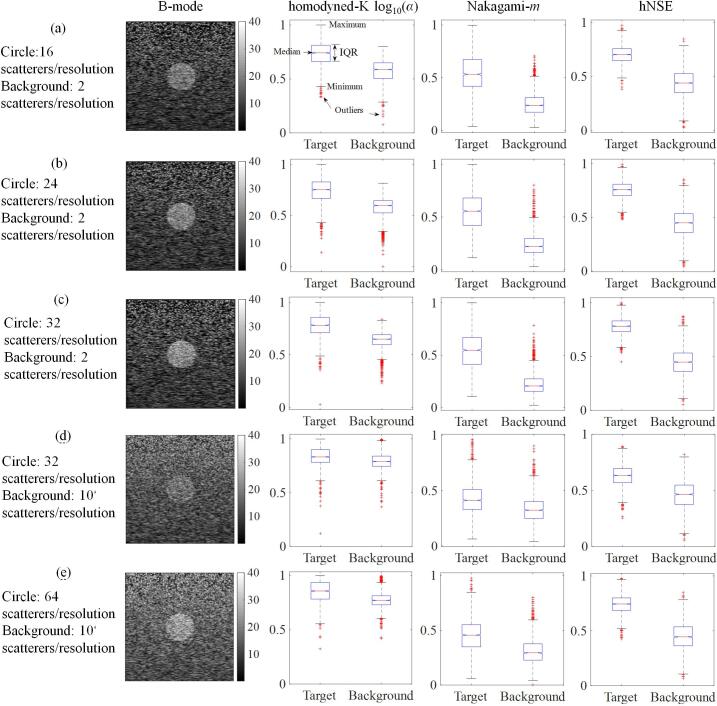
B-mode images of five different heterogeneous phantoms: (a) Circle: 16 scatterers/resolution cell, background: 2 scatterers/resolution cell; (b) Circle: 24 scatterers/resolution cell, background: 2 scatterers/resolution cell; (c) Circle: 32 scatterers/resolution cell, background: 2 scatterers/resolution cell; (d) Circle: 32 scatterers/resolution cell, background: 10 scatterers/resolution cell; (e) Circle: 64 scatterers/resolution cell, background: 10 scatterers/resolution cell, and corresponding box-plots of homodyned-K $\log_{10}(\alpha )$, Nakagami-*m*, and hNSE parametric features extracted from the circular inclusion and background regions of parametric images, respectively. IQR: interquartile range.

Fig. 5 shows B-mode images of five kinds of heterogeneous phantoms and boxplots of HK-$\log_{10}(\alpha )$, Nakagami-*m*, and hNSE parametric features extracted from the circular inclusion and background regions of parametric images, respectively, which were normalized according to Eq. (11). Each features was expressed as the median and interquartile range (IQR). The median HK-$\log_{10}(\alpha )$ was 0.74 (IQR: 0.66–0.81), 0.75 (IQR: 0.67–0.83), 0.78 (IQR: 0.71–0.86), 0.83 (IQR: 0.78–0.90), and 0.86 (IQR: 0.78–0.93) for the circular inclusions in the five kinds of heterogeneous phantoms, respectively, and the median HK-$\log_{10}(\alpha )$ was 0.59 (IQR: 0.50–0.65), 0.60 (IQR: 0.53–0.65), 0.65 (IQR: 0.60–0.69), 0.79 (IQR: 0.75–0.84), and 0.77 (IQR: 0.73–0.81) for the background regions, respectively. The median Nakagami-*m* was 0.53 (IQR: 0.42–0.67), 0.56 (IQR: 0.42–0.68), 0.55 (IQR: 0.41–0.67), 0.41 (IQR: 0.33–0.51), and 0.46 (IQR: 0.35–0.55) for the circular inclusions in the five kinds of heterogeneous phantoms, respectively, and the median *m* was 0.24 (IQR: 0.17–0.31), 0.22 (IQR: 0.16–0.30), 0.21 (IQR: 0.15–0.27), 0.32 (IQR: 0.25–0.40), and 0.29 (IQR: 0.23–0.37) for the background regions, respectively. The median hNSE was 0.70 (IQR: 0.65–0.76), 0.76 (IQR: 0.70–0.81), 0.78 (IQR: 0.73–0.83), 0.63 (IQR: 0.57–0.69), and 0.74 (IQR: 0.69–0.80) for the circular inclusions in the five kinds of heterogeneous phantoms, respectively, and the median hNSE was 0.44 (IQR: 0.35–0.53), 0.45 (IQR: 0.36–0.54), 0.45 (IQR: 0.36–0.53), 0.47 (IQR: 0.37–0.55), and 0.45 (IQR: 0.36–0.54) for the background regions, respectively. It is shown in Fig. 5 that the IQRs of HK-$\log_{10}(\alpha )$, Nakagami-*m*, and hNSE features extracted from circular target regions were substantially higher than those extracted from background regions. However, there was a certain degree of overlap between the IQRs of HK-$\log_{10}(\alpha )$ and Nakagami-*m* features extracted from circular target regions of 32 scatterers/resolution cell and those extracted from background regions of 10 scatterers/resolution cell.

**Fig. 6:**
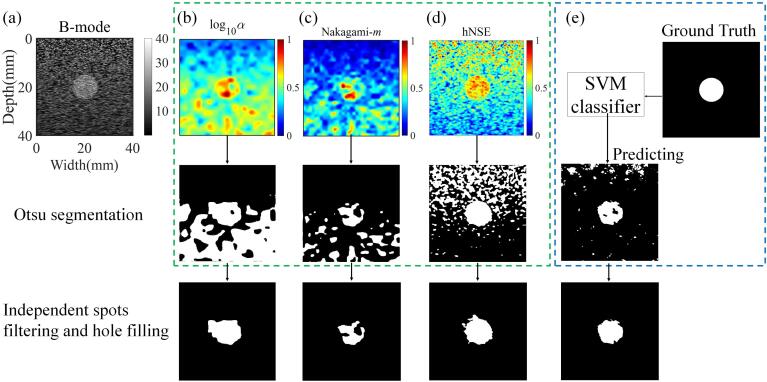
A case of heterogeneous phantom and its (a) B-mode, (b) homodyned-K $\log_{10}(\alpha )$, (c) Nakagami-*m*, and (d) horizontally normalized Shannon entropy (hNSE) parametric images with corresponding Otsu segmentation and binarized images after spots filtering and hole filling; (e) a support vector machine (SVM) classifier with [$\log_{10}(\alpha ),m$, hNSE] as the input features was used to identify the circle inclusion.

Fig. 6 shows a case of heterogeneous phantom and its B-mode, HK-$\log_{10}(\alpha )$, Nakagami-*m*, and hNSE parametric images with corresponding Otsu segmentation and binarized images after spots filtering and hole filling, comparing with a SVM classifier with the features input of [$\log_{10}(\alpha ),m$, hNSE] to identify the circular inclusions. The binarized images segmented from $\log_{10}(\alpha ),m$, and hNSE parametric imaging exhibited much interference in the background, while the binarized image predicted by SVM classifier seemed to yield least interference. The final binarized images obtained from both the hNSE parametric imaging and SVM classifier exhibited similar contour to the ground truth.

Table 1 shows the performance of the SVM classifier on the testing set of simulated heterogeneous phantoms. For different types of heterogeneous phantoms, the prediction accuracy exceeded 90%, and high sensitivity, specificity, and AUC were achieved except the case of 32 scatterers/resolution cell in the inclusion and 10 scatterers/resolution cell in the background region. Table 2 shows mean area accuracies, Dice scores, and Hausdorff distances of circular inclusion detection from five kinds of heterogeneous phantoms using single HK-$\log_{10}(\alpha )$, Nakagami-*m*, and hNSE parametric imaging with Otsu segmentation and the QUS-based multi-parameter SVM classifier. Expect for the case of 32 scatterers/resolution cell in the inclusion and 10 scatterers/resolution cell in the background, the SVM classifier exhibited highest mean area accuracies (> 93%) in identifying the circular inclusions, and was significantly higher than Nakagami-*m* and hNSE parametric imaging; moreover, the SVM classifier exhibited smaller mean Hausdorff distances (< 13) and significantly higher mean Dice scores (> 0.93) than the HK-$\log_{10}(\alpha )$, Nakagami-*m*, and hNSE parametric imaging. For the case of 32 scatterers/resolution cell in the inclusion and 10 scatterers/resolution cell in the background, the SVM classifier exhibited significantly higher mean area accuracy and Dice score, and smaller mean Hausdorff distance than the HK-$\log_{10}(\alpha )$ and Nakagami-*m* parametric imaging. These indicated that the SVM classifier has a higher accuracy in both area detection and contour detection of target inclusions than single parametric imaging with Otsu segmentation.

**Table 1:** Performance of the support vector machine classifier on the testing set of five kinds of heterogeneous phantoms (10 groups of testing sets for each type of heterogeneous phantoms). S/RC: scatterers per resolution cell.

Phantoms	Classification accuracy	Sensitivity	Specificity	AUC
Circle: 16 S/RC; Background: 2 S/RC	0.97 ± 0.03	0.90 ± 0.05	0.97 ± 0.03	0.93 ± 0.03
Circle: 24 S/RC; Background: 2 S/RC	0.98 ± 0.02	0.95 ± 0.04	0.98 ± 0.02	0.96 ± 0.02
Circle: 32 S/RC; Background: 2 S/RC	0.98 ± 0.01	0.97 ± 0.02	0.99 ± 0.01	0.98 ± 0.01
Circle: 32 S/RC; Background: 10 S/RC	0.90 ± 0.03	0.64 ± 0.01	0.91 ± 0.04	0.78 ± 0.04
Circle: 64 S/RC; Background: 10 S/RC	0.94 ± 0.03	0.95 ± 0.03	0.94 ± 0.03	0.84 ± 0.01

**Table 2:** The mean area accuracies, Dice scores, and Hausdorff distances of circular inclusion detection from five kinds of heterogeneous phantoms (10 groups of testing sets for each type of heterogeneous phantoms) using single homodyned-K $\log_{10}(\alpha )$, Nakagami-*m*, and horizontally normalized Shannon entropy (hNSE) parametric imaging with Otsu segmentation and the quantitative ultrasound based multi-parameter support vector machine (SVM) classifier. S/RC: scatterers per resolution cell. ADA: Area detection accuracy. DS: Dice score. HD: Hausdorff distances.

Phantoms	Indicators	$\log_{10}(\alpha )$	*m*	hNSE	SVM	*p*-value
Circle: 16 S/RC; Background: 2 S/RC	ADA (%)	82.59 ± 14.35	80.30 ± 11.88	71.84 ± 5.25	94.08 ± 4.34	p<0.05^2^^,^^3^^,^^4^^,^^5^p<0.001^6^
	DS	0.87 ± 0.07	0.82 ± 0.06	0.89 ± 0.04	0.93 ± 0.02	p<0.05^3^^,^^4^^,^^6^p<0.001^5^
	HD	23.79 ± 12.89	24.10 ± 7.94	30.67 ± 11.51	10.82 ± 2.20	p<0.05^3^^,^^5^p<0.001^6^
Circle: 24 S/RC; Background: 2 S/RC	ADA (%)	91.39 ± 3.40	76.98 ± 9.13	71.32 ± 7.51	93.57 ± 4.49	p<0.05^4^p<0.001^1^^,^^2^^,^^5^^,^^6^
	DS	0.89 ± 0.03	0.82 ± 0.05	0.87 ± 0.03	0.93 ± 0.01	p<0.001^1^^,^^3^^,^^5^^,^^6^
	HD	16.85 ± 5.03	22.28 ± 2.67	25.22 ± 8.30	12.43 ± 4.09	p<0.05^6^p<0.001^5^
Circle: 32 S/RC; Background: 2 S/RC	ADA (%)	89.38 ± 10.58	83.76 ± 7.52	69.53 ± 5.04	93.04 ± 2.31	p<0.001^2^^,^^4^^,^^5^^,^^6^
	DS	0.90 ± 0.05	0.86 ± 0.05	0.87 ± 0.02	0.94 ± 0.01	p<0.05^3^p<0.001^5^^,^^6^
	HD	18.24 ± 5.12	20.24 ± 6.36	23.56 ± 8.72	11.06 ± 2.33	p<0.05^3^^,^^5^^,^^6^
Circle: 32 S/RC;Background: 10 S/RC	ADA (%)	64.46 ± 13.10	44.54 ± 16.81	81.80 ± 13.35	83.76 ± 6.94	p<0.05^1^^,^^2^^,^^3^p<0.001^4^^,^^5^
	DS	0.65 ± 0.14	0.57 ± 0.17	0.86 ± 0.05	0.86 ± 0.02	p<0.05^2^^,^^3^^,^^4^^,^^5^
	HD	47.48 ± 20.64	55.71 ± 18.30	32.00 ± 11.12	25.29 ± 9.50	p<0.05^2^^,^^3^^,^^4^p<0.001^5^
Circle: 64 S/RC; Background: 10 S/RC	ADA (%)	90.79 ± 9.50	83.35 ± 9.20	76.59 ± 5.21	95.06 ± 3.47	p<0.05^1^^,^^4^^,^^5^p<0.001^2^^,^^6^
	DS	0.89 ± 0.04	0.85 ± 0.05	0.90 ± 0.02	0.94 ± 0.01	p<0.05^3^^,^^5^p<0.001^6^
	HD	18.92 ± 4.76	22.28 ± 5.19	18.49 ± 4.74	11.82 ± 2.34	p<0.05^3^^,^^6^p<0.001^5^

1 Significant difference between $\log_{10}(\alpha )$ and *m*.

2 Significant difference between $\log_{10}(\alpha )$ and hNSE.

3 Significant difference between $\log_{10}(\alpha )$ and SVM.

4 Significant difference between *m* and hNSE.

5 Significant difference between *m* and SVM.

6 Significant difference between hNSE and SVM.

**Fig. 7:**
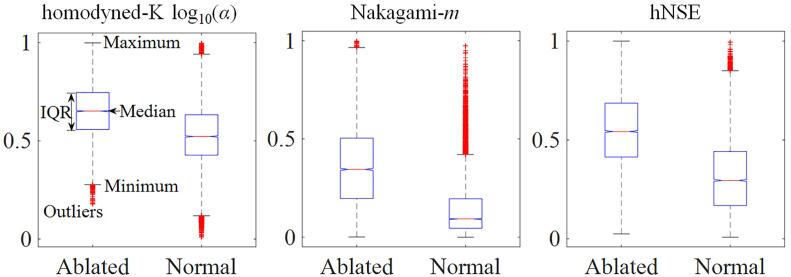
Boxplots of homodyned-K $\log_{10}(\alpha )$, Nakagami-*m*, and horizontally normalized Shannon entropy (hNSE) features extracted from thermal lesion and surrounding normal tissue regions of parametric images at 80 W, 1 min (*n* = 15); 70 W, 2 min (*n* = 15); and 60 W, 3 min (*n* = 15). IQR: interquartile range.

Fig. 7 shows the boxplots of HK-$\log_{10}(\alpha )$, Nakagami-*m*, and hNSE parametric features extracted from thermal lesions and normal tissues of parametric images, respectively. The median HK-$\log_{10}(\alpha )$ was 0.65 (IQR: 0.56–0.75) for thermal lesion regions, and 0.52 (IQR: 0.43–0.63) for normal tissues. The median Nakagami-*m* was 0.34 (IQR: 0.20–0.50) for thermal lesion regions, and 0.09 (IQR: 0.04–0.20) for normal tissues. The median hNSE was 0.54 (IQR: 0.41–0.69) for thermal lesion regions, and 0.30 (IQR: 0.17–0.44) for normal tissues. On one hand, the IQRs of Nakagami-*m* and hNSE features extracted from the thermal lesion regions were substantially higher than those extracted from normal tissues. On the other hand, although the IQR of the HK-$\log_{10}(\alpha )$ feature extracted from thermal lesion regions was basically higher than that extracted from normal tissues, there was a certain degree of overlap in the value range.

**Fig. 8:**
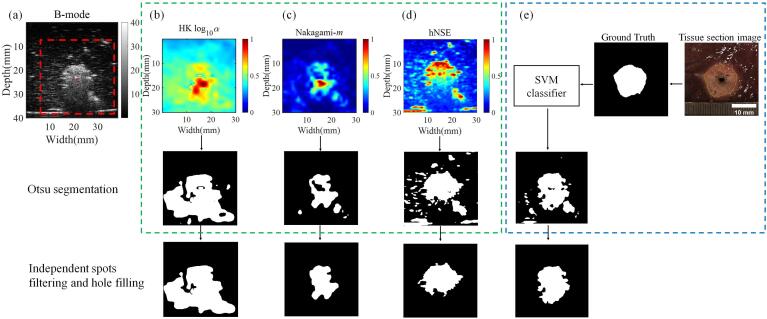
A case of microwave ablation of porcine liver *ex vivo* at 80 W, 1 min: (a) B-mode, (b) homodyned-K (HK) $\log_{10}(\alpha )$, (c) Nakagami-*m*, and (d) horizontally normalized Shannon entropy (hNSE) parametric imaging with corresponding Otsu segmentation and binarized images after spots filtering and hole filling; (e) a support vector machine (SVM) classifier with [$\log_{10}(\alpha ),m$, hNSE] as the input features was used to identify the thermal lesions, in which a 30 mm × 30 mm region of interest (ROI) selected from the tissue section image was binarized into a mask as classification ground truth. The square enclosed by dotted red line in (a) is the corresponding ROI.

Fig. 8 shows a case of MWA-induced thermal lesions in porcine livers *ex vivo* at 80 W, 1 min. It involves the B-mode [Fig. 8(a)], HK-$\log_{10}(\alpha )$ [Fig. 8(b)], Nakagami-*m* [Fig. 8(c)], and hNSE [Fig. 8(d)] parametric imaging with corresponding Otsu segmentation and binarized images after spots filtering and hole filling. Fig. 8(e) shows a SVM classifier with [$\log_{10}(\alpha ),m$, hNSE] as the input features to identify the thermal lesions, in which a 30 mm × 30 mm region of interest (ROI) selected from the tissue section image of the ablated porcine liver was binarized into a mask as the classification ground truth. The ablated regions in the binarized images segmented from HK-$\log_{10}(\alpha )$ and *m* parametric imaging were significantly larger and smaller than the ground truth, respectively, whereas the ablated region in the binarized images segmented from hNSE imaging and predicted by SVM classifier was more similar to the ground truth. Meanwhile, the binarized image predicted by the SVM classifier exhibited less interference in the background than that obtained from hNSE imaging.

Table 3 shows the performance of the SVM classifier on the testing set of porcine liver *ex vivo* induced by MWA at 60–80 W, 1–3 min (*n* = 15). The mean classification accuracy, sensitivity, specificity, and AUC of the testing samples were 0.85, 0.84, 0.85, and 0.84, respectively. Table 4 shows the mean area accuracies, Dice scores, and Hausdorff distances of thermal lesions detection from porcine liver *ex vivo* induced by MWA (*n* = 15) using single HK-$\log_{10}(\alpha )$, Nakagami-*m*, and hNSE parametric imaging with Otsu segmentation and the QUS-based multi-parameter SVM classifier. The SVM classifier achieved the highest mean area accuracy and Dice score, which were significantly higher than those obtained from HK-$\log_{10}(\alpha )$, Nakagami-*m*, and hNSE parametric imaging. Meanwhile, the mean Hausdorff distance obtained from the SVM classifier was significantly smaller than that obtained from HK-$\log_{10}(\alpha )$, Nakagami-*m*, and hNSE parametric imaging.

**Table 3:** Performance of the support vector machine classifier on the testing set of porcine liver microwave ablation *ex vivo* (15 groups of testing sets).

Power and duration	Classification accuracy	Sensitivity	Specificity	AUC
80 W, 1 min (*n* = 5); 70 W, 2 min (*n* = 5); 60 W, 3 min (*n* = 5)	0.85 ± 0.06	0.84 ± 0.08	0.85 ± 0.07	0.84 ± 0.05

**Table 4:** The mean area accuracies, Dice scores, and Hausdorff distances of thermal lesion detection from porcine livers *ex vivo* induced by microwave ablation (15 groups of testing sets) using single homodyned-K $\log_{10}(\alpha )$, Nakagami-*m*, and horizontally normalized Shannon entropy (hNSE) parametric imaging with Otsu segmentation and the quantitative ultrasound based multi-parameter support vector machine (SVM) classifier. S/RC: scatterers per resolution cell. ADA: Area detection accuracy. DS: Dice score. HD: Hausdorff distances.

Power and duration	Indicators	$\log_{10}(\alpha )$	*m*	hNSE	SVM	*p*-value
80 W, 1 min (*n* = 5);70 W, 2 min (*n* = 5);60 W, 3 min (*n* = 5)	ADA (%)	84.59 ± 22.91	65.14 ± 12.00	74.19 ± 18.82	89.10 ± 7.33	p<0.05^2^^,^^3^^,^^4^^,^^6^p<0.001^1^^,^^5^
	DS	0.56 ± 0.09	0.71 ± 0.08	0.70 ± 0.08	0.77 ± 0.05	p<0.001^1^^,^^2^^,^^3^^,^^5^^,^^6^
	HD	111.02 ± 34.92	78.26 ± 34.22	80.38 ± 25.05	46.38 ± 22.31	p<0.05^1^^,^^2^^,^^5^^,^^6^p<0.001^3^

1 Significant difference between $\log_{10}(\alpha )$ and *m*.

2 Significant difference between $\log_{10}(\alpha )$ and hNSE.

3 Significant difference between $\log_{10}(\alpha )$ and SVM.

4 Significant difference between *m* and hNSE.

5 Significant difference between *m* and SVM.

6 Significant difference between hNSE and SVM.

## 4 Discussion

In this study, a QUS-based SVM classifier was proposed for tissue characterization, specifically for characterization of tissue scatterer distribution patterns. The proposed method was compared with HK-$\log_{10}(\alpha )$, Nakagami-*m*, and hNSE parametric imaging through five types of phantom simulations and MWA of porcine livers *ex vivo*. Furthermore, the feasibility in detecting thermal lesions by the proposed method was evaluated.

Currently, there are several ultrasound imaging techniques for detecting thermal lesions or ablation zones, such as ultrasound elastography [64], contrast-enhanced ultrasound (CEUS) [65], QUS [23], and deep learning methods [66]. Among them, heat-induced gas bubbles generated during the ablation can cause artifacts in ultrasound elastography of ablated regions [67], so ultrasound elastography is suggested for the evaluation of thermal lesions after ablation. Pohlman et al. [68] attempted to characterize post-ablation zones using electrode displacement elastography for 13 patients with hepatocellular carcinoma or liver metastasis and compared the binarized images of coagulation zones with clinical-standard-of-care segmentation, yielding an average Dice score of 0.88. CEUS provides highly sensitive visualization of blood flow in tissues because of the utility of intravascular microbubble contrast agents [69], which makes it the primary clinical method for evaluating the treatment effect after ablation. Liu et al. [70] used CEUS to quantitatively evaluate the thermal lesions of the livers of 10 young living swine induced by RFA; compared with histopathology, CEUS had an accuracy of 81.4%, a sensitivity of 83.3%, and a specificity of 76.9%. Methods of deep learning overcomes the difficulty of finding and computing the features related to lesions, but it needs a large amount of training data, which puts strict requirements on data acquisition and labeling. Zhang et al. [66] proposed an ultrasound imaging method based on a convolutional neural network (CNN) architecture for the detection and monitoring of thermal lesions induced by MWA in porcine livers, where RF data were acquired as the input layer of trained network, and segmentation images based on CNN were reconstructed to display the predicted thermal lesions, yielding an AUC of 0.89 and a Dice score of 0.87. In addition, single QUS envelope statistics imaging methods have been proposed to detect MWA-induced thermal lesions. In our previous work [11], [22], the mean coagulation area detection accuracies of 20 cases of MWA-induced porcine liver *ex vivo* based on Nakagami-*m* and HK α and *k* parametric imaging combined with PAX were 89.26%, 89.47%, and 85.27%, respectively. The H-scan-based HK contrast-weighted summation α and *k* parametric imaging combined with Otsu segmentation, spots filtering, and hole filling achieved mean MWA coagulation area detection accuracies over 87% and 66%, respectively, with mean Dice scores being over 0.73 and 0.57, respectively [11]. Li et al. [45] proposed hNSE imaging for MWA-induced thermal lesion detection, with an accuracy of 79.03%, a sensitivity of 79.19%, a specificity of 78.72%, and an AUC of 0.87.

The QUS method quantitatively extracts acoustic characteristic parameters from ultrasound backscattered echo signals. HK, Nakagami, and entropy parametric imaging is an important group of QUS imaging techniques, which uses the statistics in the backscattered echo signals to reflect changes of tissue microstructures. HK and Nakagami imaging methods are model-based techniques, which requires that the envelope of the backscattered signals obey a specific distribution model, whereas entropy imaging is a non–model-based method, which has better adaptability to the data. Note that prior to estimating parametric models, one could classify pixels into a few labels (corresponding to distinct echo envelope distribution) in order to meet the assumption of a single distribution on each sample set [71]. Typically, a normal liver parenchyma can be considered as a collection of considerable scatterers [72]. Most of the liver volume is occupied by hepatocytes, which contribute to diffuse scattering [73]. The hepatocyte is very small; it is approximately cubical, with a side length of 20–30 μm and a volume of 3.4 × $10^{-6}$ ${\mathrm{mm}}^{3}$ [74]. Since the strength of ultrasound backscattering depends on the size of scatterers, the hepatocytes may be considered as weak scatterers exhibiting low echogenicities. During MWA, the rapid increase of temperature around the tip of ablation needle led to coagulative necrosis of the tissue in this region, where protein denaturation, cytoplasm disintegration, organelle dysfunction, nucleus and cell membranes destruction, and the presence of vapor bubbles all occurred [37], [38]. These might act as additional acoustic scatterers causing the increases of scatterer concentrations in the ablation zone. The HK α and Nakagami-*m* parameters that determine the statistical distribution of ultrasonic backscattered envelope were used to characterize the scatterer concentrations, since both α and *m* vary with the scatterer concentration. Moreover, ablation led to a higher uncertainty of backscattered signals in ablated tissues than in normal tissues. Entropy accurately captured and characterized the uncertainty and complexity of backscattered signals in ablated tissues which carried information of microstructure changes through calculation of PDHs.

Although the feasibility of deep learning in monitoring MWA has been demonstrated [66], the acquisition of large amounts of training data and labels becomes a major obstacle to its clinical application. In contrast, conventional machine learning algorithms, such as SVMs, can use less training data and labels to achieve the construction of classification models, and may be more explainable. The use of QUS parameters as input features of machine learning models has been studied in the lesion diagnosis and tumor classification [71], [75], [76]. To the best of our knowledge, this paper is the first to use QUS parameters as the input features of the SVM classifier for MWA thermal lesion detection. In addition, our study found that the proposed method exhibited better performance in identifying coagulation zones than the single parametric imaging (HK-$\log_{10}(\alpha )$, Nakagami-*m*, or hNSE), which indicated that the combination of QUS-based multi-parameter features effectively enhanced the discrimination of ablated tissues. The SVM classifier can classify the image pixel by pixel after ablation, outperforming the method of Otsu image segmentation for single parametric images, which can effectively avoid the impact of the segmentation algorithm on the detection accuracy of the coagulation zones.

In the phantom experiments, we simulated five types of heterogeneous phantoms with higher concentrations in the circular inclusions and lower concentrations in the background media. When the concentration of the weak scattering background media was 2 scatterers/resolution cell and the inclusion concentrations were 16, 24, and 32 scatterers/resolution cell, the effectiveness of the proposed method in characterizing different scatterer distribution patterns was validated. We used scattering media of 10 scatterers/resolution cell (i.e., the echo amplitude follows the Rayleigh distribution) to represent normal liver tissues because the hepatocytes were explained to act as scattering sources at the Rayleigh scattering level [77]. Since we did not know how many scatterers are added to the ablation zone due to MWA-induced bubbles, circular inclusions of 32 and 64 scatterers/resolution cell were used to represent the coagulation zone. It was found in Table 1 that the greater the difference of scatterer concentrations between the inclusion and background regions, the better the classification performance of the SVM classifier.

The optimal input feature vector, i.e., [$\log_{10}(\alpha ),m$, hNSE], was determined by experiments. For instance, we compared the accuracy, sensitivity, specificity, and AUC obtained using different feature combinations as inputs of the SVM classifier in the MWA experiments of porcine liver *ex vivo* (*n* = 15), and the results are shown in Table 5. Overall, the three-parameter feature combination [$\log_{10}(\alpha ),m$, hNSE] and four-parameter feature combination [$\log_{10}(\alpha ),k,m$, hNSE] both exhibited the best performance of classification. The results showed that there was no need to add the HK *k* parameter into the input feature of the SVM classifier. Moreover, it has been demonstrated that *k* parametric imaging was not as good as α parametric imaging in characterizing thermal lesions [11], [24]. This might be due to that the *k* parameter represents the ratio of the coherent to diffuse signal, whereas the characterization of ablation zones was more related to the scatterer concentration and arrangement, which could be better described by α,m, or hNSE. The feature vector [$\log_{10}(\alpha ),m$, hNSE] used in this study yielded better performance than the combinations of two features or one single feature. Besides, when the ratio of the scatterer concentration of inclusion-to-background was smaller, the performance of the proposed method would be lower (Table 1, Table 2). Since the input features of SVM, namely, $m,\log_{10}(\alpha )$, and hNSE, needed to distinguish different scattering media by characterizing scatterer concentrations and signal uncertainty, when the difference between the concentrations of the background and inclusion media is small, the $m,\log_{10}(\alpha )$, and hNSE values extracted from these two regions may be nearly the same, which may not act as effective input features for SVM models. Therefore, the proposed method is suggested to characterize tissue scatterer distribution patterns for different scatterer concentrations.

**Table 5:** The accuracy, sensitivity, specificity, and AUC obtained by using different feature combinations as inputs of the SVM classifier in MWA experiments of porcine liver *ex vivo* (15 groups of testing sets). AUC: area under the operating characteristic curve; SVM: support vector machine; MWA: microwave ablation.

**Input feature(s)**	**Accuracy**	**Sensitivity**	**Specificity**	**AUC**
$\log_{10}(\alpha ),k,m$, hNSE	0.85 ± 0.06	0.84 ± 0.10	0.85 ± 0.08	0.84 ± 0.05
$\log_{10}(\alpha ),m$, hNSE	0.85 ± 0.06	0.84 ± 0.08	0.85 ± 0.07	0.84 ± 0.05
$\log_{10}(\alpha ),m$	0.85 ± 0.07	0.71 ± 0.11	0.88 ± 0.09	0.79 ± 0.05
$\log_{10}(\alpha )$, hNSE	0.82 ± 0.10	0.74 ± 0.15	0.85 ± 0.14	0.79 ± 0.05
*m*, hNSE	0.80 ± 0.04	0.83 ± 0.18	0.81 ± 0.08	0.82 ± 0.06
$\log_{10}(\alpha )$	0.74 ± 0.14	0.64 ± 0.24	0.75 ± 0.22	0.69 ± 0.06
*m*	0.85 ± 0.06	0.75 ± 0.10	0.88 ± 0.07	0.80 ± 0.07
hNSE	0.78 ± 0.10	0.68 ± 0.21	0.81 ± 0.16	0.75 ± 0.05

This study has limitations. First, the simulated phantoms did not consider acoustic attenuation and noise, as the primary goal of this study was to validate the feasibility of multimodality QUS envelope statistics imaging based machine learning models (specifically, SVMs) in enhancing the capability of characterizing scatterer distribution patterns. Note that the echo envelope distribution depended on the settings of focused transducer [78] and the total attenuation of the intervening tissues, even when considering non–model-based methods, which also affected the parameter estimation. Besides, in order to keep the settings of the phantom simulations consistent with or close to those of the MWA experiments, the element width of the transducer we set in simulations was higher than half of the central wavelength, which might cause clutter and degrade the parameter estimation. The 100 pixels randomly selected from each simulated phantom acquisition are not guaranteed to be uncorrelated since some may be close to one another. Second, the types of simulated inclusions and the types of MWA experiments of porcine liver *ex vivo* are limited. In addition, only MWA experiments of porcine liver *ex vivo* was carried out to investigate the feasibility of the proposed method. *In vivo* animal experiments and clinical studies may be conducted in future work. Third, outliers in Nakagami parameter estimates in particular may be due to using the moments-based estimator rather than lower-variance methods like the maximum likelihood estimator. The better Nakagami parameter estimator can be used to replace the moments-based estimator in future work. In addition, only HK-$\log_{10}(\alpha )$, Nakagami-*m*, and hNSE were combined as the input features for the SVM classifier. Other parameter estimators may be used and more QUS parameters can be studied as the input of the machine learning model in future work.

The typical central frequency of the curved-array ultrasound transducer commonly used for clinical *in vivo* liver imaging is around 3.5 MHz. The central frequency of the linear-array ultrasound transducer used in this study (phantom simulations and *ex vivo* experiments) was 7.5 MHz. A higher central frequency corresponds to a smaller size of the resolution cell, which can make the ultrasound system more sensitive to changes in scatterer concentrations, and may provide a more optimistic performance of the SVM. However, a lower central frequency of the 3.5-MHz curved-array transducer corresponds to a larger penetration depth, which is inconvenient for MWA experiments of porcine liver *ex vivo* because the thickness of porcine liver samples *ex vivo* is limited. We used a linear-array transducer with a central frequency of 7.5 MHz to conduct *ex vivo* experiments, whose scanning depth was 4 cm. In order to be consistent with the configuration of the *ex vivo* experiments, the central frequency of the ultrasound transducer in the phantom simulations was also set to 7.5 MHz. Nevertheless, in accordance with the experimental results of this study, we hypothesize that for the 3.5-MHz curved-array transducer, the SVM model also can enhance the capability of scatterer distribution pattern characterization over the single ultrasound envelope statistics imaging method. This hypothesis needs to be validated in future work.

## 5 Conclusion

In this paper, a QUS-based multi-parameter SVM classifier was proposed for characterizing scatterer distribution patterns and for detecting MWA-induced thermal lesions. Through phantom simulations and MWA experiments of porcine livers *ex vivo*, the proposed method was demonstrated to outperform the HK-$\log_{10}(\alpha )$, Nakagami-*m*, hNSE parametric imaging in identifying the higher-scatterer-concentration zones and MWA-induced coagulation zones.

## CRediT authorship contribution statement

**Sinan Li:** Conceptualization, Methodology, Software, Investigation, Writing – original draft. **Po-Hsiang Tsui:** Methodology, Software, Data curation, Writing – original draft, Funding acquisition. **Weiwei Wu:** Formal analysis, Visualization, Investigation, Funding acquisition. **Zhuhuang Zhou:** Methodology, Software, Writing – review & editing, Supervision, Funding acquisition. **Shuicai Wu:** Resources, Writing – review & editing, Supervision, Funding acquisition.

## Declaration of competing interest

The authors declare that they have no known competing financial interests or personal relationships that could have appeared to influence the work reported in this paper.
